# Exogenous Nitric Oxide Enhances Disease Resistance by Nitrosylation and Inhibition of *S*-Nitrosoglutathione Reductase in Peach Fruit

**DOI:** 10.3389/fpls.2020.00543

**Published:** 2020-05-20

**Authors:** Zifei Yu, Jixuan Cao, Shuhua Zhu, Lili Zhang, Yong Peng, Jingying Shi

**Affiliations:** ^1^Key Laboratory of Food Processing Technology and Quality Control in Shandong Province, College of Food Science and Engineering, Shandong Agricultural University, Tai’an, China; ^2^College of Chemistry and Material Science, Shandong Agricultural University, Tai’an, China

**Keywords:** *Prunus persica*, nitric oxide, *S*-nitrosoglutathione reductase, brown rot, nitrosylation

## Abstract

Nitric oxide (NO), a signaling molecule, participates in defense responses during plant–pathogen interactions. *S*-Nitrosoglutathione (GSNO) is found to be an active intracellular NO storage center and regulated by *S*-nitrosoglutathione reductase (GSNOR) in plants. However, the role of GSNOR in NO-induced disease resistance is not clear. In this research, the effects of NO and GSNOR inhibitor (N6022) on the defense response of harvested peach fruit to *Monilinia fructicola* infection were investigated. It was found that the disease incidence and lesion diameter of peach fruits were markedly (*P* < 0.05) reduced by NO and GSNOR inhibitor. However, the expression of *GSNOR* was significantly inhibited (*P* < 0.05) by NO only during 2–6 h. Analyses using iodo-TMT tags to detect the nitrosylation sites of GSNOR revealed that the sulfhydryl group of the 85-cysteine site was nitrosylated after NO treatment in peach fruit at 6 and 12 h, suggesting that exogenous NO enhances disease resistance via initial inhibition of gene expression and the *S*-nitrosylation of GSNOR, thereby inhibiting GSNOR activity. Moreover, NO and GSNOR inhibitor enhanced the expression of systemic acquired resistance (SAR)-related genes, such as pathogenesis-related gene 1 (*PR1*), nonexpressor of *PR1* (*NPR1*), and TGACG-binding factor 1 (*TGA1*). These results demonstrated that *S*-nitrosylation of GSNOR protein and inhibition of GSNOR activity contributed to the enhanced disease resistance in fruit.

## Introduction

Peach [*Prunus persica* (L.) Batsch] fruit is rich in nutrients that provide favorable growth conditions for pathogenic bacteria. In addition, due to the high temperature and environmental humidity during the picking period of the peach fruit, the infection and growth of pathogenic bacteria are accelerated, causing a large amount of decay of the peach fruit, resulting in huge economic losses. Brown rot caused by *Monilinia fructicola* is severely destructive to stone fruits, involving in cherries, plums, and peaches (Hu et al., 2011).

At present, cold storage and chemical fungicides are widely used to inhibit brown rot development of peach fruit. Studies have shown that cold storage can effectively control *M. fructicola* disease by delaying spore germination (Sommer, 1985). However, peach fruit is very sensitive to low temperature, resulting in chilling injury during cold storage. Chemical fungicides can inhibit brown rot (Adaskaveg et al., 2005), but long-term use of chemicals may induce several problems such as fungicide resistance, chemicals residues on fruits, and environmental pollution. Therefore, the development of safe and effective methods is necessary to control brown rot in stone fruit.

Induced resistance based on biotic or abiotic activation of certain cellular defense responses is considered a sustainable strategy to inhibit pathogen invasion and reduce postharvest decay (Romanazzi et al., 2016). Nitric oxide (NO) is an active molecule with high fat solubility that can diffuse rapidly through the cell membrane. NO is also a gaseous radical. NO and NO-derived molecules are collectively referred to as reactive nitrogen species (RNS) (Corpas et al., 2007). Less is known about the role of NO-derived molecules in the interactions of plants with pathogens (Chaki et al., 2009). However, the function of NO as an important RNS in plants has been extensively studied (Corpas et al., 2007; Besson-Bard et al., 2008). NO is involved in various physiological processes in plants, including seed germination, growth, development, maturation, senescence, and stress response (Arasimowicz and Floryszak-Wieczorek, 2007). Moreover, during plant–pathogen interactions, NO participates in defense responses (Romero-Puertas et al., 2004). Salicylic acid (SA)-mediated activation of signaling pathways is an important manifestation of NO-induced resistance in plants (Domingos et al., 2015). Studies have shown that exogenous NO treatment has obvious inhibitory effects on pathogens including *Penicillium expansum*, *Botrytis cinerea*, and *Colletotrichum gloeosporioides* of postharvest fruits (Zheng et al., 2011; Lai et al., 2014; Hu et al., 2019). However, mechanisms of disease resistance induced by NO in harvested peach fruit are not well understood.

Stamler (1994) first proposed the concept of protein nitrosylation modification, that is, the NO group is covalently bonded to the cysteine (Cys) residue of the protein to produce *S*-nitrosothiol (SNO). Nitrosylation can influence the structure, activity, and function of target proteins (Stamler et al., 2001; Chen et al., 2006), thereby affecting the corresponding signal transduction pathways in the cell. Numerous studies confirm that *S*-nitrosylation plays a wide range of roles in different pathological and physiological processes (Hess and Stamler, 2012). Recently, it is found that NO may accomplish long distance signal transduction by protein nitrosylation in plants (Skelly and Loake, 2013; Kulik et al., 2015). *S*-nitrosoglutathione reductase (GSNOR), classified as the alcohol dehydrogenase family, which exists in all species from bacteria to humans, is an important regulatory protein in NO turnover (Liu et al., 2001). Feechan et al. (2005) found that GSNOR, associated with protein nitrosylation in *Arabidopsis*, plays a critical role in disease resistance. GSNOR has attracted more and more attention as an important regulator of nitrosylation of proteins.

*S*-Nitrosoglutathione (GSNO) is a storage site for the biological activity of NO. GSNOR regulates the levels of GSNO and other *S*-nitrosothiols (SNOs) and protein nitrosylation in eukaryotic cells (Liu et al., 2001). As a key factor in plant disease resistance response, GSNOR causes changes in intracellular redox status by regulating GSNO content, which in turn leads to *S*-nitrosylation of defense-related proteins (Sakamoto et al., 2002; Dìaz et al., 2003; Lee et al., 2008). Post-translational modification of these proteins can cause different defense responses in plants (Rustérucci et al., 2007; Kwon et al., 2012). However, the study of GSNOR in post-harvest fruits has not been reported. Therefore, it is of great significance to elucidate the role of GSNOR in the disease resistance of post-harvest peach fruit.

## Materials and Methods

### Fruit Materials and Treatments

Peach fruit (cultivar “Zhonghuashoutao”) were collected from Yiyuan, Shandong Province, China, and selected for uniform size and no mechanical damage. According to previous methods, peach fruit were soaked in either deionized water (served as control), NO solution (15 μmol L^–1^; diluted with a saturated NO solution made from NO gas), or GSNOR inhibitor [N6022, bought on MCE official website (MedChemExpress)^1^ ] solution (60 μmol L^–1^), respectively, for 20 min and dried at room temperature (Gu et al., 2014). A 3 mm × 3 mm × 3 mm wounded site was made on each fruit and inoculated with 20 μl of 1 × 10^5^ spores ml^–1^ of brown rot spore suspension. Then, the fruits were stored in a constant temperature and humidity chamber [23°C, 85–90% relative humidity (RH)] to observe the disease development in peach fruit. The disease incidence and lesion diameter were measured at 48 and 72 h, respectively. Six repetitions are set for each treatment, and each repetition contained 30 peaches. Samples of three peach fruits at each time point were taken out from each repetition and cut into small pieces. Then, the pieces were frozen with liquid nitrogen, grinded into power, and stored at −80°C. Moreover, samples of three fruits of each repetition were taken at 0, 6, 12, 24, 48, and 72 h for RNA and protein extraction, enzyme assay, and the content of SNOs, GSNO, glutathione of reduced state (GSH), glutathione disulfide (GSSG), and endogenous NO measurement.

### Measurement of GSNOR and GR Activity in Different Treated Peach Fruit

Glutathione reductase (GR) activity was measured according to Knorzer et al. (1996). One half gram peach powder was homogenized in 4 ml of 50 mM phosphate-buffered saline (PBS) [pH 7.0, containing 20% (*v*/*v*) glycerol, 2 mM DL-dithiothreitol (DTT), 2 mM ethylene diamine tetraacetic acid (EDTA), 2% polyvinylpyrrolidone (PVP)]. The supernatant obtained after centrifugation at 20,000 × *g* for 30 min at 4°C was used to determine the enzyme activity. The reaction system included 100 μl supernatant, 3 ml reaction liquid [pH 7.5, containing 50 mM Tris–HCl, 0.5 mM GSSG, 5 mM MgCl_2_, and 0.2 mM reduced nicotinamide adenine dinucleotide phosphate (NADPH)]. The changes in absorbance at 340 nm were determined.

*S*-nitrosoglutathione reductase activity was measured following the means of Sakamoto et al. (2002). One half gram peach powder was extracted with 3 ml assay mixture containing 50 mM HEPES (pH 8.0), 20% (*v*/*v*) glycerol, 10 mM MgCl_2_, 1 mM EDTA, 1 mM ethylene glycol-bis(2-aminoethylether)-N,N,N′,N′-tetraacetic acid (EGTA), 1 mM benzamidine, and 1 mM ε-aminocaproic acid at 4°C and then centrifuged at 16,000 × *g* for 15 min. Three hundred microliters of supernatant was incubated in 3 ml assay mixture containing 20 mM Tris–HCl (pH 8.0), 0.5 mM EDTA, and 0.2 mM NADH, and the GSNO was mixed to a final concentration of 400 mM to initiate the reaction.

### Measurement of SNOs and NO Levels in Differently Treated Peach Fruits

The SNO content was measured according to Frungillo et al. (2013) with some modifications. One half gram peach powder was homogenized in 4 ml of 100 mM phosphate buffer (pH 7.2, containing 100 mM EDTA, 100 mM EGTA), after which centrifugation was carried out at 20,000 × *g* for 30 min at 4°C to obtain the supernatant. One milliliter supernatant was reacted with solution I [containing 1% sulfanilamide, 0.1% *N*-(1-naphthyl) ethylene-diamino dihydrochoride] or solution II [containing 1% sulfanilamide, 0.1% *N*-(1-naphthyl) ethylene-diamino dihydrochoride, 2 mM HgCl_2_] in a ratio of 1:1. After incubating in the dark for 10 min, the reaction solution was centrifuged at 12,000 × *g* for 5 min. The SNO content in plants was quantified by determining the absorbance difference between solutions II and I at 540 nm. The absorbance at 540 nm was determined using different concentrations of GSNO instead of the enzyme solution, and a standard curve was prepared. The content of SNOs in the plants was calculated according to the value of the standard curve.

The NO content was performed according to the means of Shi et al. (2015). One half gram peach powder was extracted in 3 ml 50 mM glacial acetic acid buffer [pH 3.6, containing 4% (*w*/*v*) zinc acetate], then centrifuged at 10,000 × *g* for 15 min at 4°C. Equal volumes of extraction and Griess reagent (Sigma-Aldrich, St Louis, United States) were mixed and reacted at 25°C for 30 min. The optical density (OD) value at 540 nm was determined.

### Measurement of the Content of Endogenous GSNO, GSSG, and GSH in Peach Fruit

The contents of GSNO, GSSG, and GSH were measured according to Hodges and Forney (2000) with minor modifications. One half gram peach powder was dissolved with 5 ml of 5% (*w*/*v*) 5-sulfosalicylic acid and then centrifuged at 12,000 × *g* for 15 min at 4°C. Solution A (containing 100 mM Na_2_HPO_4_⋅7H_2_O, 40 mM Na_2_HPO_4_⋅H_2_O, 1.8 mM 5,5′-dithiobis-(2-nitrobenzoic acid) (DTNB), 15 mM EDTA, and 0.04% (*w*/*v*) bovine serum albumin (BSA)] and solution B (including 1.0 mM EDTA, 50 mM imidazole, and 0.02% (*w*/*v*) BSA] were prepared and then adjusted to pH 7.2. The GSNO content was measured by mixing 800 μl solution A, 640 μl solution B, 800 μl of 1:25 dilution of extract in 0.5 M K_2_HPO_4_ buffer (pH 7.0), and 160 μl of 3.0 mM NADPH. The changes in the OD value of the mixed solution at 412 nm were recorded. The GSH content was measured by adding 300 μl of 10 U mol^–1^ glutathione reductase (GR) (100 U) to the above reaction system for 1 min, and the change of OD value at 412 nm for 5 min was measured. The measurement method of GSSG was as follows. 1.0 ml extract was diluted into 1:10 in 0.5 M K_2_HPO_4_ buffer (pH 6.5, containing 20 μl 2-vinylpyridine) at 25°C for 1 h. Then, 400 μl of solution A and 320 μl of solution B was added, and the change in OD value was determined at 412 nm. The standard curve of GSNO, GSSG, and GSH was prepared, and the content of GSNO, GSSG, and GSH in the sample was calculated according to the standard curve.

### Transcript Analyses of *GR*, *GSNOR*, and Defense-Related Gene Expression by Real-Time Quantitative PCR

Total RNA extraction was conducted from samples at various time intervals. One hundred milligrams of the sample was taken, and the RNAprep Pure Polysaccharide Polyphenol Plant Total RNA extraction kit (DP441) produced by Tiangen (Shanghai, China) was used for extraction of the total RNA. The CWBIO HiFiScript cDNA Synthesis Kit (CW2596) was use to synthesize complementary DNA (cDNA) by incubating at 42°C for 50 min, then at 85°C for 5 min. The primers of *GR*, *GSNOR*, *PR1*, *NPR1*, and *TGA1* were designed based on their genetic sequences (Table 1). *TEF2* (TC3544) and *tubulin-α* (DY650410) from peach fruit were used as reference genes (Tong et al., 2009). Real-time (RT-PCR) was performed using of the CWBIO UltraSYBR Mixture (CW0957) kit with reaction volumes of 25 μl.

**TABLE 1:** Primers used for real-time qPCR analysis.

Gene name	Primer sequences	Tm (°C)	Product
			size (bp)
***GR***	F: CCTTCAATCTGGGCTGTA	55	158
	R: ATTGGTGGCTGGGAAA		
***GSNOR***	F: TGTTCATGATGTTAGTGTTGCG	58	121
	R: TGATTCTACTTTTGCCGTGTTC		
***PR1***	F: TCTAACACTTGTGCCGATGAC	58	126
	R: ATAGTTGCACCCGATGAAGG		
***NPR1***	F: CAGATGATGTGAACTTGTGAA	55	71
	R: GTAAGCCGCAGCATAATG		
***TGA1***	F: GCCTCAGCATCAATGATAGTTG	58	105
	R: TGCTTCTTGGTCATACTTGCTA		

### Nitrosylation Site Detection

Some experimental methods involved in the detection of nitrosation sites mainly refer to Gong and Shi (2019) and Xie et al. (2016) with slight modifications. The experimental methods and steps were as follows.

#### Protein Extraction From Peach Fruit

The peach fruit treated with NO, inoculated with *M. fructicola* then stored for 4, 6, 12, and 24 h, was ground into powder in liquid nitrogen. The sample with lysis buffer [including 8 M urea, 100 mM triethylammonium bicarbonate buffer (TEAB, Sigma-Aldrich, St Louis, MI, United States), 50 mM Iodoacetamide (IAM, Sigma-Aldrich, St Louis, MI, United States), 1% Protease Inhibitor Cocktail (Merck Millipore, Billerica, MA, United States), and 1% Triton X-100] added was sonicated three times on ice. The lysate was incubated at room temperature without light for 30 min, and then, centrifugation was performed at 20,000 × *g* at 4°C for 10 min to remove the remaining debris. Finally, cold 20% TCA was used to precipitate the protein at −20°C for 2 h. The precipitate obtained by centrifugation was washed three times with cold acetone. HES buffer [50 mM TEAB, 1 mM EDTA, and 0.1% sodium dodecyl sulfate (SDS)] was used to redissolve the protein. The protein concentration was measured according to the instructions of the BCA kit (Beyotime Biotechnology, Shanghai, China).

#### Iodo-TMT Labeling

One milligram protein per treatment was redissolved in 1 ml HES buffer and processed with the iodo-TMT kit (Thermo Fisher Scientific, Waltham, MA, United States). In short, iodo-TMT reagent dissolved in 10 μl MS grade methanol was mixed to the redissolved protein solution; then, 20 μl of 1 M sodium ascorbate was added and mixed briefly. The mixed solution was incubated in the dark at 37°C for 2 h. The reaction was quenched by the addition of 40 μl of 0.5 M DTT (20 mM final concentration) and incubated in darkness at 37°C for 15 min.

#### Trypsin Digestion

Six volumes of prechilled (−20°C) acetone were used to precipitate the mixed labeled protein at −20°C for at least 2 h and then centrifuged to discard the supernatant. The protein precipitate was dissolved in 8 M urea after washing with cold acetone three times. The protein solution after reduction with 5 mM DTT for 30 min at 56°C was alkylated with 11 mM IAM for 15 min at room temperature without light. The protein samples were diluted with 100 mM TEAB, and the final urea concentration was below 2 M. Trypsin was added at a trypsin-to-protein mass ratio of 1:50 overnight, and a second digestion was performed at a mass ratio of 1:100 trypsin to protein for 4 h. Strata X C18 SPE column (Phenomenex, Torrance, CA, United States) was used for desalting of trypsin-digested peptides. Finally, the peptide was dried under vacuum.

#### HPLC Fractionation

Fractionation of the samples was carried out by high pH reverse-phase HPLC (EASY-nLC 1000, Thermo Fisher Scientific, Waltham, MA, United States) applying 300 Extend C18 column (5 μM particles, 4.6 mM ID, and 250 mM length; Agilent Technologies, Santa Clara, CA, United States). First, the peptides were dissociated into 80 fractions with a gradient of 2–60% acetonitrile (ACN) in 10 mM NH_4_HCO_3_ (pH 10) over 80 min. Then, the peptides were combined into four fractions and vacuum dried.

#### Affinity Enrichment

Tryptic peptides were dissolved in TBS buffer (pH 7.5, 150 mM NaCl, 250 mM Tris–HCl) and incubated with prewashed anti-TMT antibody beads (Thermo Fisher Scientific, Waltham, MA, United States) at 4°C overnight to enrich cysteine nitrosylation peptides The beads were cleaned once with disinfected deionized water after three times with TBS buffer. The peptide bound to the beads was eluted with elution buffer (0.4% TFA, 50% ACN). The eluted peptides were mixed and vacuum dried. The peptides obtained after C18 ZipTips (Millipore, Boston, MA, United States) washing were analyzed by liquid chromatography tandem mass spectrometry (LC-MS/MS).

### LC-MS/MS Analysis

Refer to the means of Yang et al. (2018) with minor modifications. The peptides were resuspended in solvent A (containing 0.1% FA and 2% ACN) and separated using an EASY-nLC 1000 UPLC system (Thermo Fisher Scientific, Waltham, MA, United States). The gradient of solvent B (containing 0.1% FA and 98% ACN) included an increase from 6 to 25% over 26 min, 25 to 40% in 8 min, and climbing to 80% in 3 min, then remaining at 80% for the last 3 min. The flowrate was maintained at 400 nl min^–1^. The peptides were injected into the NSI source for ionization and then analyzed using tandem mass spectrometry (MS/MS) on an Q Exactive^TM^ Plus (Thermo Fisher Scientific, Waltham, MA, United States) coupled online to the ultraperformance liquid chromatography (UPLC).

### Statistical Analysis

The MaxQuant search engine (v.1.5.2.8) was used to analysis the resulting MS/MS data. The UniProtKB *Prunus persica* (sequences: 28234. Version: 2016.5.30) database was connected to the reverse decoy database to search for tandem mass spectra. IodoTMT-6plex was selected as the quantification method. The fold-change threshold was set when peptides with quantitative ratios over 1.2 or under 1/1.2 are considered significant. Intensive bioinformatic analyses were then carried out to annotate those quantifiable lysine acetylated targets in response to NO solution treatment, including Gene Ontology (GO) annotation, subcellular localization, Kyoto Encyclopedia of Genes and Genomes (KEGG) pathway annotation, etc. Based on the results, further studies following the quantitative cysteine nitrosylation analysis were suggested. Three repetitions were used in nitrosylation measurement. The compilation and mapping of experimental data were performed using Microsoft Excel 2013 and Sigma Plot 10.0 software. IBM SPSS version 20.0 was used for statistical analysis, and significant (*P* < 0.05) level test was performed using least significant difference (LSD) test.

## Results

### Effect of NO and GSNOR Inhibitor on Disease Incidence and Lesion Diameter of Peach Fruit Inoculated With *M. fructicola*

**FIGURE 1:**
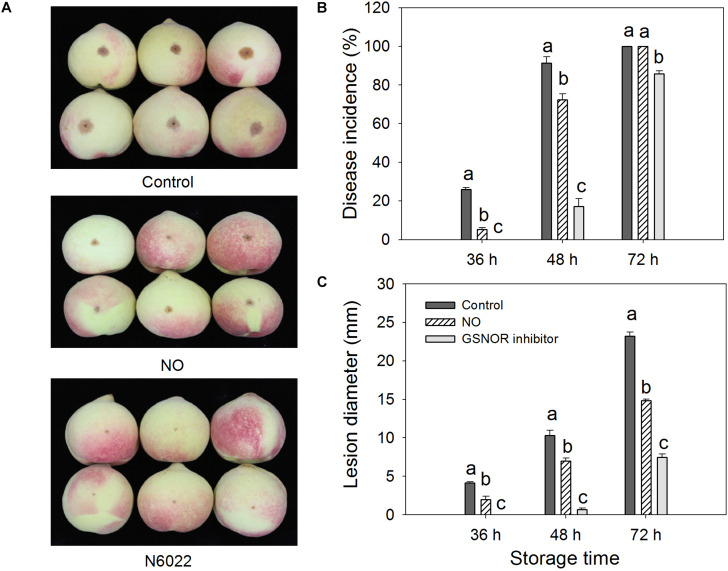
Effect of nitric oxide (NO) or *S*-nitrosoglutathione reductase (GSNOR) inhibitor treatment on the **(A)** disease development (at 48 h after inoculation), **(B)** disease incidence, and **(C)** lesion diameter of peach fruit after inoculation with *M. fructicola* spore suspension (1 × 10^5^ spores ml^–1^) and then stored at 23°C. Bars represent standard deviations of the means (*n* = 6). Different letters represent significant differences (*p* < 0.05) according to least significant difference (LSD) test.

The disease incidence and lesion diameter of peach fruit inoculated with *M. fructicola* were significantly (*P* < 0.05) suppressed by NO and GSNOR inhibitor (N6022), and the inhibitory effect of GSONR inhibitor on *M. fructicola* was better than that of NO solution treatment (Figure 1). At 36 h, the disease incidence of the control fruit was approximately 15%, and the average lesion diameter was 2.3 mm, however, the disease symptom was not present on GSNOR inhibitor-treated fruit. During the investigated period, the lesion development on NO-treated or GSNOR inhibitor-treated fruit was lower than that on the control (Figure 1A). Moreover, 36 and 48 h, the NO and GSNOR inhibitor-treated fruit exhibited significantly (*P* < 0.05) lower disease incidence than the control (Figure 1B). The lesion diameter on the control fruit was 2.1, 1.5, and 1.6 times of NO-treated fruit, and even at 72 h, the lesion diameter on the control fruit was almost 3.1 times that of GSNOR inhibitor-treated peach fruit (Figure 1C). These results indicate that treatment with NO and GSNOR inhibitor reduces disease incidence and the extent of lesions. GSNOR inhibitor was the most effective at inhibiting brown rot development.

### Effect of NO and GSNOR Inhibitor on the Activity and Gene Expression of GR and GSNOR in Peach Fruit

**FIGURE 2:**
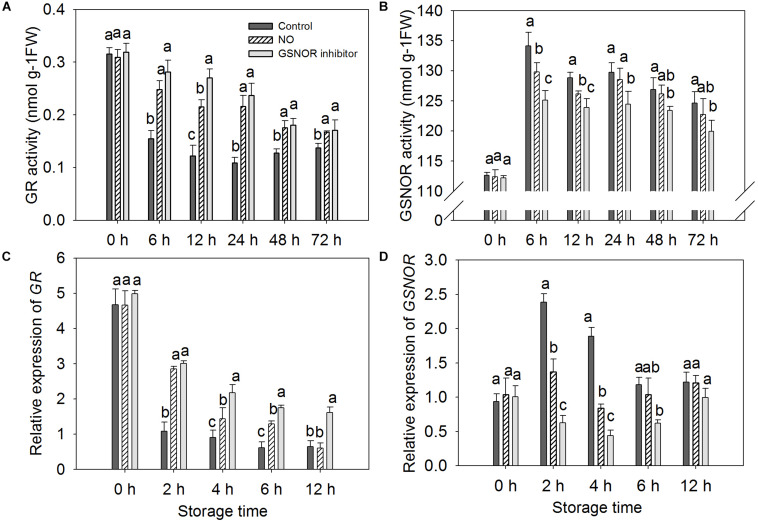
Effect of nitric oxide (NO) or *S*-nitrosoglutathione reductase (GSNOR) inhibitor treatment on the activity of **(A)** glutathione reductase (GR) and **(B)** GSNOR and the **(C)** gene expression of *GR* and **(D)** *GSNOR* of peach fruit storage at 23°C. Vertical bars represent the standard deviation of the means (*n* = 6). Different letters represent significant differences (*P* < 0.05) according to least significant difference (LSD) test.

The activity and gene expression of GR and GSNOR were measured in peach fruit after inoculation with *M. fructicola* (Figure 2). Compared with the control, NO and GSNOR inhibitor obviously (*P* < 0.05) enhanced the activity of GR. The enhancement effect of NO on GR activity was very obvious at 6–24 h, but the GR activity was slightly different from the control at 48 and 72 h. The effect of GSNOR inhibitor on GR activity was more obvious than that of NO and GR activity, showing a downward trend at 0–72 h (Figure 2A). The activity of GSNOR showed a tendency of increase first and then decrease. Both NO and N6022 inhibited the activity of GSNOR, and the inhibitory effect of N6022 treatment was stronger than that of NO treatment (Figure 2B). Furthermore, higher levels of *GR* expression were observed in both NO-treated and GSNOR inhibitor-treated fruit than the control (Figure 2C). *GSNOR* expression in peach fruit treated with NO and GSNOR inhibitor was obviously (*P* < 0.05) lower than the control at 2–6 h. The expression of *GSNOR* in all three treatments stabilized at 12 h. GSNOR inhibitors have the most marked inhibitory effect on *GSNOR* gene expression (Figure 2D).

### Determination of the S-Nitrosylation Site of GSNOR in Peach Fruit After Treated With NO

**FIGURE 3:**
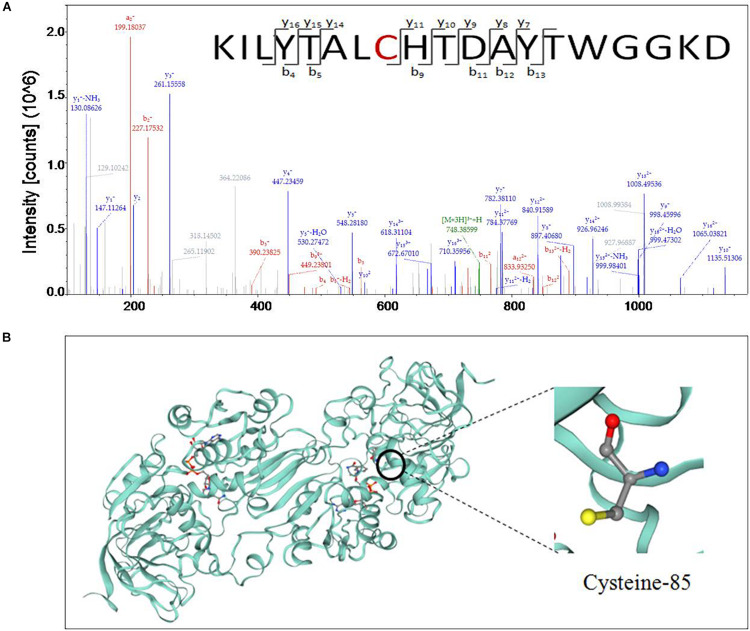
**(A)** Cys-85 of *S*-nitrosoglutathione reductase (GSNOR) was identified as an *S*-nitrosylated residue by site-specific proteomic mass spectrometry, and **(B)** 3D structure model of GSNOR protein of peach was predicted in Uniprot. Illustrated in the figure is the cysteine located at position 85 of the amino acid sequence.

Nitric oxide-treated and non-treated peach fruits inoculated with *M. fructicola* were quantitatively measured for nitrosylation using iodo-TMT tags. The sulfydryl in Cys-85 of GSNOR was identified as an *S*-nitrosylated residue in peach fruit treated with NO at 6 and 12 h (Figure 3A). The peptide sequences of nitrosylation was KILYTALCHTDAYTWGGKD. To get the 3D structural model of GSNOR, find the GSNOR (M5VJ61) information page on the UniProt official website and click M5VJ61 under “Structure” (Figure 3B). From the structure of the model, GSNOR is a dimer containing two subunits, and its ligands include two zinc ion and two nicotinamide adenine dinucleotide.

**FIGURE 4:**
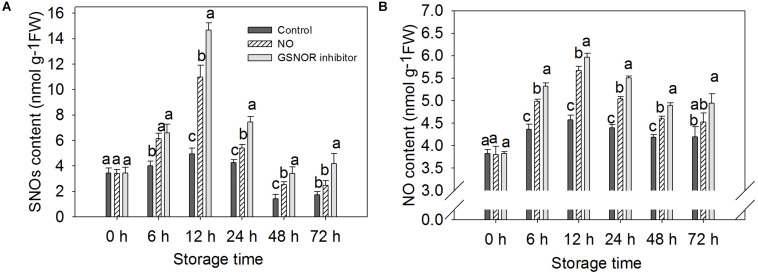
Effect of nitric oxide (NO) or *S*-nitrosoglutathione reductase (GSNOR) inhibitor treatment on the content of **(A)** SNOs and **(B)** NO of peach fruit storage at 23°C. Vertical bars represent the standard deviation of the means (*n* = 6). Different letters represent significant differences (*P* < 0.05) according to least significant difference (LSD) test.

The content of endogenous NO and SNOs increased from 0 to 12 h and then decreased in both treated and the control fruit from 12 to 48. At 72 h, the content increased slightly but did not change much. The change in endogenous NO content is similar to SNOs (Figure 4). Treatment with NO and GSNOR inhibitor increased the levels of SNOs and NO in peach fruit compared to the control (Figures 4A,B). GSNOR inhibitor treatment had a stronger effect on the content of SNOs and NO than exogenous NO treatment.

### Effect of Different Treatments on the Content of GSH, GSSG, and GSNO

**FIGURE 5:**
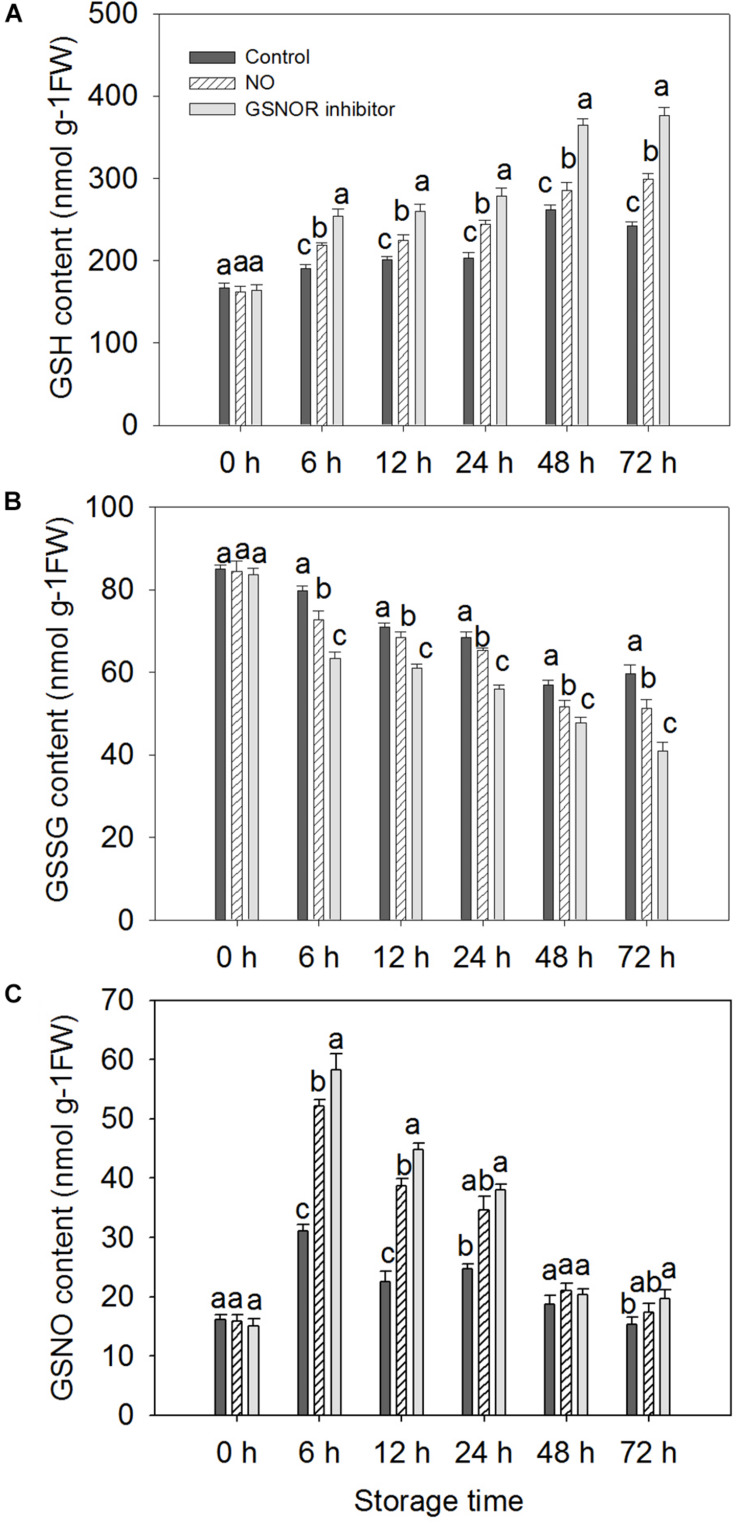
Effect of nitric oxide (NO) or *S*-nitrosoglutathione reductase (GSNOR) inhibitor treatment on the level of **(A)** GSH, **(B)** GSSG, and **(C)** GSNO of peach fruit during storage at 23°C. Vertical bars represent the standard deviation of the means (*n* = 6). Different letters represent significant differences (*P* < 0.05) according to least significant difference (LSD) test.

The level of GSH increased from 0 to 72 h in the treated fruit. However, GSH levels in control fruits increased from 0 to 48 h and decreased at 72 h (Figure 5A). GSNOR inhibitor treatment enhanced GSH level maximum, NO treatment was the second, and the GSH level of the control peach fruit was relatively low (Figure 5A). The change in GSSG content is opposite to that of GSH. GSSG content showed a downward trend within 0–48 h after inoculation with *M. fructicola*. The GSSG content of the control fruits increased at 72 h, but a decrease in GSSG levels was still found in the treated peach fruit (Figure 5B). The level of GSNO increased significantly at 0–6 h, and then decreased. It can be seen from the figure that NO and GSNOR inhibitor treatment could obviously (*P* < 0.05) increase the level of GSNO during 0–24 h. However, GSNO content in the three treatment fruits had no significant difference (*P* > 0.05) at 48 h. At 72 h, the content of GSNO in peach fruits treated with GSNOR inhibitor was higher than the other two groups (Figure 5C).

### Effect of NO and GSNOR Inhibitor on the Expression of Several Defense-Related Genes in Peach Fruit

**FIGURE 6:**
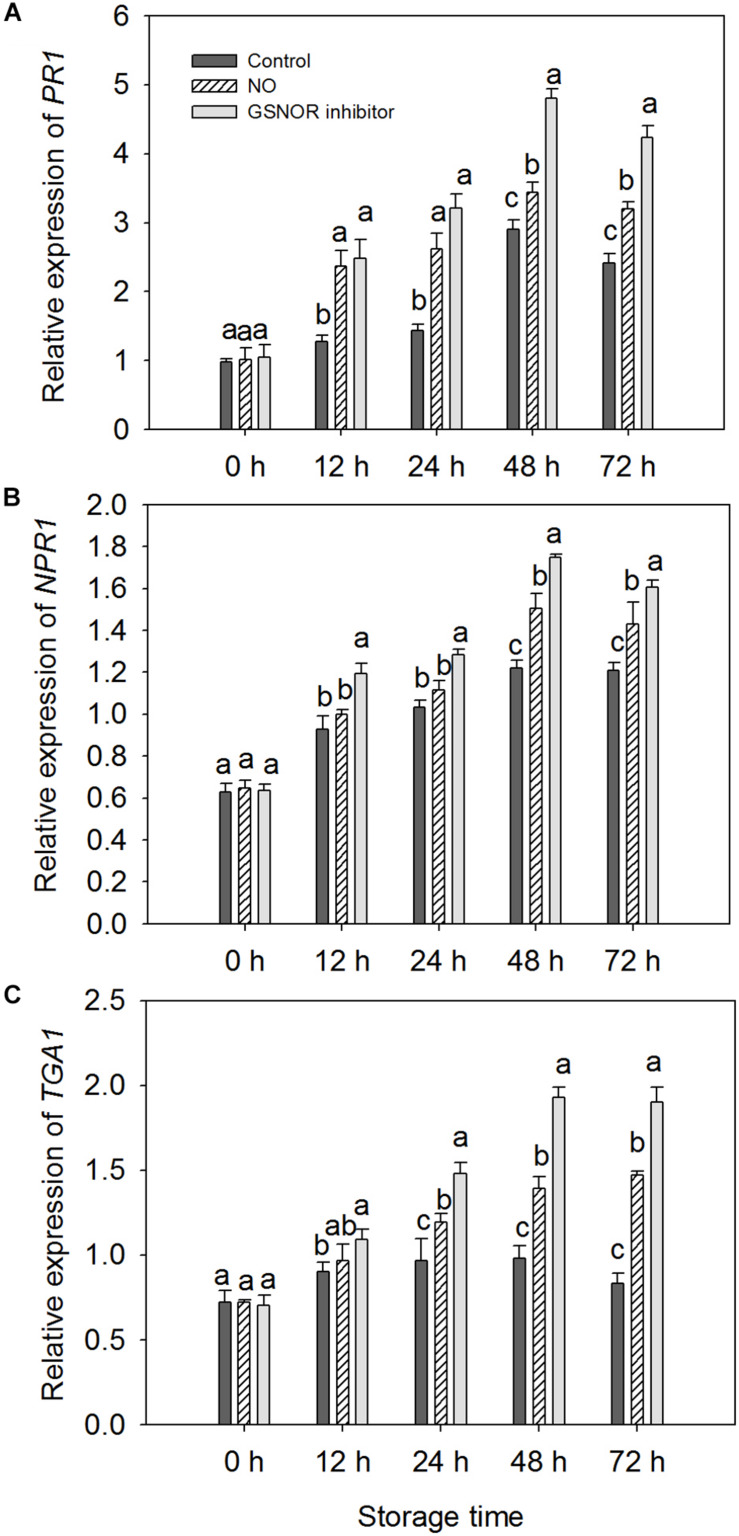
Effects of nitric oxide (NO) or *S*-nitrosoglutathione reductase (GSNOR) inhibitor treatment on **(A)** *PR1*, **(B)** *NPR1*, and **(C)** *TGA1* gene expression, determined by real-time qPCR. All experiments were run in triplicate with different complementary DNAs (cDNAs) synthesized from six biological replicates. Vertical bars represent the standard deviation of the means (*n* = 6). Different letters represent significant differences (*P* < 0.05) according to least significant difference (LSD) test.

The expressions of three defense-related genes in peaches pretreated with NO and GSNOR inhibitor were investigated by RT-PCR. NO and GSNOR inhibitor increased the expression levels of *PR1*, *NPR1*, and *TGA1* genes (Figure 6). The expression trend of *PR1* was similar to that of *NPR1*, gradually increasing during 0–48 h. It decreases when it reaches the maximum at 48 h (Figures 6A,B). The expression level of *TGA1* in the control fruit was relatively stable, but NO and GSNOR inhibitor significantly (*P* < 0.05) induced *TGA1* gene expression from 24 to 72 h (Figure 6C).

## Discussion

Nitric oxide involves participating in plant responses to biotic and abiotic stresses as an important signaling molecule (Mur et al., 2006; Carreras and Poderoso, 2007). It is reported that NO has a direct and effective inhibitory effect on a variety of microorganisms (Schairer et al., 2012). However, in our previous studies, low concentration of NO solution could effectively restrict disease development but had no significant inhibition against *M. fructicola in vitro* (Gu et al., 2014). In recent years, more and more studies have shown that NO has obvious inhibitory effects on post-harvest diseases. Treatment of tomato fruit with L-arginine, a precursor of NO, inhibits the expansion of lesion diameter caused by *B. cinerea* (Zheng et al., 2011). In apple fruit, NO donor sodium nitroprusside (SNP) treatment could inhibit virulence of *P. expansum*, causing obviously lower disease incidence and smaller lesion diameter compared to the water treatment (Lai et al., 2014). Hu et al. (2019) showed that NO could significantly increase the resistance of pitaya fruit to *C. gloeosporioides* after harvest, which is consistent with the results observed on the present result in peach fruit.

The pyrrole group located in N6022 is an efficient GSNOR inhibitor with potent inhibitory ability (Sun et al., 2011). The inhibitory effect of N6022 on GSNOR activity has been shown to be safe and effective in animal models of asthma and inflammatory disease. As a tight bonding inhibitor, N6022 is currently under application for early clinical research in humans (Green et al., 2012). However, it has not been used in postharvest fruits. The present results show that the GSNOR inhibitor N6022 reduces disease incidence significantly (*P* < 0.05) and enhances the resistance of peach fruit to brown rot while reducing the activity of GSNOR of peaches.

*S*-nitrosoglutathione reductase regulates the levels of GSNO and SNOs in eukaryotic cells by specifically recognizing and degrading GSNO that can be decomposed into GSSG and ammonia (NH3) (Liu et al., 2001). As the most abundant of endogenous intracellular SNOs, GSNO is considered as a potential NO storage site or transport center in the cell (Butler and Rhodes, 1997; Mayer et al., 1998; Liu et al., 2001). It can transfer NO to the target protein to change the function of the protein (Liu et al., 2001). The present results show that NO-treated fruit had markedly (*P* < 0.05) higher content of GSNO than the control, which contributed to promoting the nitrosylation of the target protein of GSNOR (Figures 3A, 5C). Studies have shown that when plants are infected with pathogenic bacteria, the content of SNOs will increase, suggesting that SNOs play a vital role in signal transduction and host defense (Feechan et al., 2005; Chaki et al., 2009). The present results showed that the content of SNOs increased at 0–12 h, and both NO and GSNOR inhibitor treatments promoted the production of SNOs, which was helpful for the improvement of disease resistance of peach fruit (Figure 4A). It is reported that the concentration of NO in the cells increased, and NO could bound to GSH to form GSNO under stress conditions (Liu et al., 2001). Under the catalysis of GR, the oxidized form of glutathione (GSSG) can be readily converted to the reduced state (GSH). GSH is a major intracellular antioxidant that eliminates reactive oxygen species (ROS) (Macdonald et al., 2003). GR maintains a high GSH/GSSG ratio by reducing GSSG to play a key role in the antioxidant defense process (Foyer and Noctor, 2005; Patel and Patra, 2015). The present result showed that exogenous NO and N6022 treatments promoted the degradation of GSSG by increasing the activity of GR, thereby increasing the content of GSH, thus improving the antioxidant capacity of peach fruit.

The accumulation of endogenous NO after inoculation of *Rhizoctonia solani* in cucumber plant may be due to a decrease in GSNOR activity (Nawrocka et al., 2019). Our study also found the content of endogenous NO increased when GSNOR activity was inhibited by exogenous NO and GSNOR inhibitor (Figure 4B). Romero-Puertas et al. (2008) has identified some *S*-nitrosylated proteins in *Arabidopsis thaliana* during defense response of the plant, which shows that protein nitrosylation plays an indispensable role in the process of defense reaction. Our nitrosylation modification analysis showed that the GSNOR protein in the NO-treated peach fruit was nitrosylated, which indicates that exogenous NO may participate in the disease resistance response by nitrosylation modification of this protein. As GSNOR inhibitor or NO treatment reduced the activity of GSNOR in peach fruit, the degrading activity of GSNOR on GSNO declined, which leads to GSNO and other SNOs accumulation in peach fruit. Studies have shown that nitrosation of cysteine results in reduced GSNOR activity in *Arabidopsis*, budding yeast, and humans (Guerra et al., 2016). Similar results were shown in this research. Therefore, it can be speculated that NO induces nitrosylation of cysteine-85 in GSNOR, which results in lower GSNOR activity in peach fruit. Lee et al. (2008) found that transcriptional levels or protein abundance levels of GSNOR was not significantly (*P* < 0.05) regulated under stress conditions in *Arabidopsis*, suggesting that some mean of redox regulation via cysteine modification may be a mechanism to control its activity. This study indicated that the relative gene expression of *GSNOR* in the NO-treated peach fruit was not markedly (*P* > 0.05) different from the control after 6 h, but the GSNOR activity was markedly (*P* < 0.05) inhibited (Figure 2). Therefore, the nitrosylation of GSNOR may be another main regulation mode besides the transcriptional control that occurred to its enzyme activity changes.

*S*-nitrosoglutathione reductase is a highly conserved protein rich in cysteine. Through analysis, it is found that the GSNOR homology between peach and *Arabidopsis* reached 84.17% (Supplementary Figure S1). Xu et al. (2013) analyzed the structure of *Arabidopsis* GSNOR protein and found that Cys-10, Cys-271, and Cys-370 of AtGSNOR are solvent accessible and can provide conditions for post-translational modification. In addition, it was suggested that these three residues may be conserved in regulating the activity of GSNOR. Moreover, Cys-10, Cys-271, and Cys-370 of AtGSNOR have been identified as *S*-nitrosylated residues (Guerra et al., 2016; Zhan et al., 2018). However, Cys-85 of GSNOR of peach was found to be an *S*-nitrosylated residues in our identification results. Sequence analysis of GSNOR revealed that Cys-85 corresponds to Cys-47 in *Arabidopsis thaliana*, *Antrodia camphorata*, and tomato (Supplementary Figures S2, S3). At the same time, Cys-85 residue was also found to be conserved in other plant GSNORs. Huang et al. (2009) found that Cys-47 of *A. camphorata* is closely related to the enzyme activity of GSNOR. This further indicates the possible role of Cys-85 nitrosylation in the control of protein activity.

Pathogenesis-related gene 1 was first identified from *Nicotiana tabacum* infected with tobacco mosaic virus (TMV) in the 1970s (Van Loon and Van Kammen, 1970). After that, similar *PR1* proteins from many species of both mono- and dicotyledonous plants have been reported successively (Ohshima et al., 1987; Tornero et al., 1997; Maleck et al., 2000; Liu and Xue, 2006; Mitsuhara et al., 2008). Studies have confirmed that *PR1* is capable to protect plants against abiotic stress (Rauscher et al., 1999; Cutt et al., 1989). Klessig et al. (2000) demonstrated that NO can participate in the induction of *PR1* gene expression. Inhibition of *Arabidopsis GSNOR1* expression enhances *Arabidopsis* resistance to *Peronospora parasitica* and promotes systemic acquired resistance (SAR) and *PR1* expression (Rustérucci et al., 2007). Our research has yielded similar results, which indicates that GSNOR may have the parallel role in post-harvest fruits. Gene expression of *PR1* is upregulated when GSNOR is inhibited by NO and GSNOR inhibitors. NPR1 and TGA1 are pivotal redox-controlled regulators of SAR in plants. NO could promote the ability of NPR1 to increase DNA binding activity of TGA1 (Lindermayr et al., 2010). When the SA-mediated defense response is activated, changes in intracellular redox status will lead to the revivification of NPR1 to its active monomeric form. The *NPR1* monomers interacts with the reduced form of TGA1 to promote the binding of TGA1 to the activation sequence-1 (as-1) element of the promoter region of defense proteins. Studies have confirmed that this interaction promotes the DNA combing activity of TGA1 to the as-1 of the *PR-1* gene to motivate its expression (Després et al., 2003; Pieterse and Van Loon, 2004; Lindermayr et al., 2010). Lindermayr et al. (2010) showed that GSNO protects TGA1 from oxygen-mediated modifications and promotes the combing of TGA1 to the as-1 when NPR1 is present. From our results, it can be seen that both NO and GSNOR inhibitor treatment increased the content of GSNO and endogenous NO, and the expressions of *NPR1* and *TGA1* were obviously higher than the control. Therefore, treatment of peach fruit by NO and GSNOR inhibitors may promote SAR by activating the SA signaling pathway, thereby improving the disease resistance of peach fruit.

*S*-nitrosoglutathione reductase is critical for GSNO and SNOs homeostasis, as well as protecting against nitrosative stress. GSNOR activity is also a major regulator of intracellular GSNO and SNO levels, playing a key role in regulating plant resistance (Liu et al., 2001; Lindermayr et al., 2006). Salicylic acid (SA), as an immune activator in plants, is controlled by *GSNOR1* in its synthesis and signaling transduction, so the regulation of GSNOR activity is closely related to plant disease resistance (Feechan et al., 2005; Malik et al., 2011). In this study, GSNOR inhibitor N6022 was used for soaking postharvest peach fruits that were later inoculated with *M. fructicola*. It was found that N6022 could significantly (*P* < 0.05) inhibit the disease spot development of peach fruit. Moreover, the expression of *GSNOR* was decreased while the expression of *PR1* was significantly increased, and the content of endogenous SNOs and glutathione (GSH) in fruits was increased. The results indicate that partial inhibition of GSNOR activity can lead to increase in intracellular SNOs levels, accumulation of GSH, and enhancement of fruit defense capacity.

**FIGURE 7:**
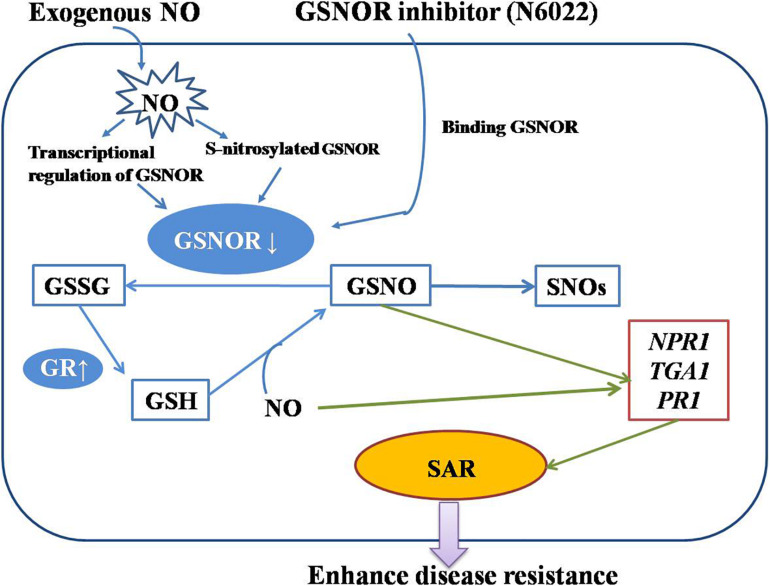
A diagram of the action mechanism of exogenous nitric oxide (NO) and N6022 on improving disease resistance of peach fruits made according to our research.

Plant defense response is a complex network involving the transmission of multiple hormones and signaling molecules. Our research mainly revealed an important defense mechanism for GSNOR to regulate the level of nitrosation and promote the production of SAR by regulating the levels of GSNO and NO (Figure 7). This provides a new orientation for probing the defense mechanism of peach fruit against *M. fructicola* and lays a foundation for in-depth research on the function of GSNOR in harvested fruit. However, it is unclear whether other signaling pathways have an effect on this mechanism, which requires more extensive research.

## Conclusion

In conclusion, the results of this study point to the key role of GSNOR in the potential molecular mechanisms of peach fruit resistant to brown rot stress. Studies have found that NO and N6022 have a good inhibitory effect on peach fruit brown rot. They inhibit the GSNOR activity and cause the accumulation of SNO, GSNO, and endogenous NO in peach fruit, and increase the GSH accumulation through enhancing GR activity, leading to the enhancement of antioxidant and defense capabilities of peach fruit. In addition, NO and N6022 activate the SA signaling pathway by promoting the expression of SAR-related genes (such as *PR1*, *NPR1*, and *TGA1*), which are also important components of peach fruit defense mechanism. Interestingly, Cys-85 residue of GSNOR was identified as an *S*-nitrosylated residue in the NO-treated peach fruit. This has not been reported in heretofore studies. It is speculated that this may be related to GSNOR enzyme activity, but further research is needed to support this conclusion. Collectively, presented data uncover that inhibition of GSNOR activity has a positive role in peach fruit response to *M. fructicola* infection.

## Data Availability Statement

The raw data supporting the conclusions of this article will be made available by the authors, without undue reservation, to any qualified researcher.

## Author Contributions

JS designed the experiments. ZY and JC conducted the experiments and analyzed the data. ZY conceived the manuscript. JS, YP, LZ, and SZ provided the valuable advice and revised the manuscript.

## Conflict of Interest

The authors declare that the research was conducted in the absence of any commercial or financial relationships that could be construed as a potential conflict of interest.

## Footnotes

1 https://www.medchemexpress.cn/
