# Supplementary figures and images for: Exogenous Nitric Oxide Enhances Disease Resistance by Nitrosylation and Inhibition of S-Nitrosoglutathione Reductase in Peach Fruit
Source: Front Plant Sci. 2020 May 20;11:543. doi: 10.3389/fpls.2020.00543 (PMC7326068; doi:10.3389/fpls.2020.00543)

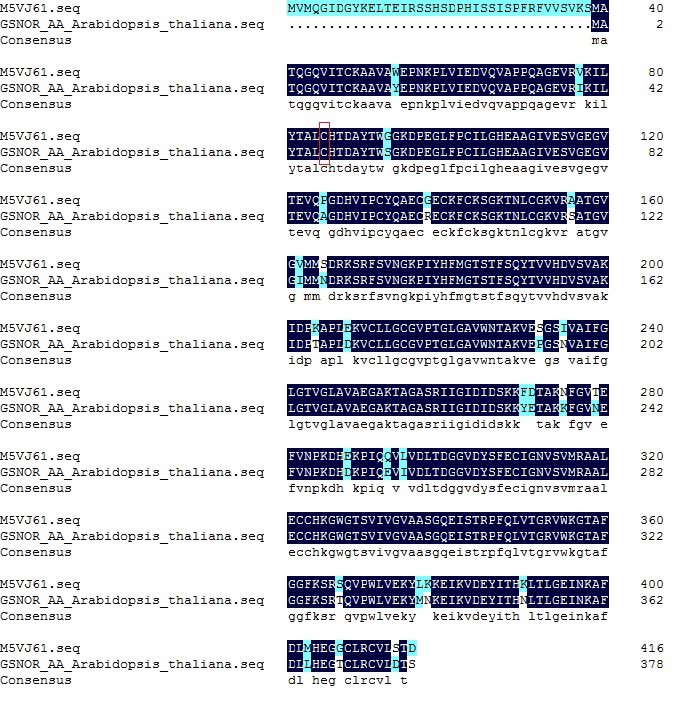

Supplement: Supplementary file 1 [file Image_1.jpeg]

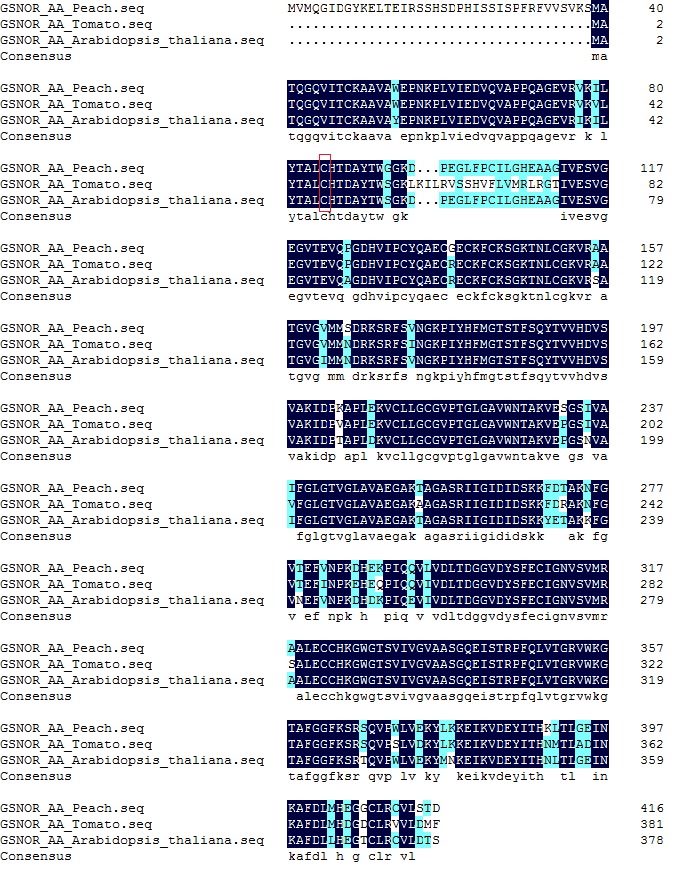

Supplement: Supplementary file 2 [file Image_2.jpeg]

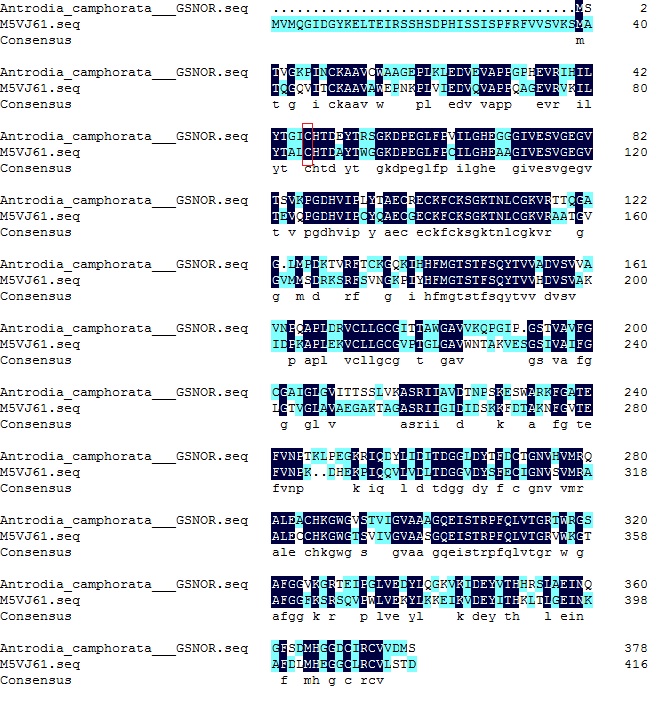

Supplement: Supplementary file 3 [file Image_3.jpeg]
