# Supplementary material for: Exogenous Nitric Oxide Enhances Disease Resistance by Nitrosylation and Inhibition of S-Nitrosoglutathione Reductase in Peach Fruit
Source: Front Plant Sci. 2020 May 20;11:543. doi: 10.3389/fpls.2020.00543 (PMC7326068; doi:10.3389/fpls.2020.00543)
Supplement: Supplementary file 4 [file Table_1.docx]

Supplementary Figure S1 GSNOR amino acid sequence alignment of peach and Arabidopsis thaliana

Supplementary Figure S2 Amino acid sequence alignment of peach, tomato and Arabidopsis thaliana

Supplementary Figure S3 GSNOR amino acid sequence alignment of Antrodia camphorata and peach
